# Susceptibility variation to different entomopathogenic nematodes in *Strategus aloeus* L (Coleoptera: Scarabaeidae)

**DOI:** 10.1186/s40064-015-1412-x

**Published:** 2015-10-16

**Authors:** A. Gómez, A. Sáenz-Aponte

**Affiliations:** Laboratory for Biological Control, Plant Biology and Productive System Group, Department of Biology, School of Sciences, Pontificia Universidad Javeriana, Bogotá, Colombia

**Keywords:** Little bull, Chiza, Oil palm, Steinernematidae, Heterorhabditidae, Larvae, Biological control

## Abstract

*Strategus aloeus* L (Coleoptera: Scarabaeidae), known as “Little bull” or oil palm “chiza” is a limiting pest in palm plantation in Cesar Colombia. Its management is based on pesticide use or old palm removal in renewal lots. Therefore, other alternatives are being sought out. Entomopathogenic nematodes isolated from the Colombian Andean region were evaluated. Under laboratory conditions *S. aloeus* third instar larvae exposure to 160 infective juveniles (IJs) per/cm^2^*Steinernema* sp3 JCL027, *S. feltiae* SCT125, *S. websteri* JCL006, *S. colombiense* SNI0198, *Heterorhabditis bacteriophora* HNI0100, *H. bacteriophora* HASA702, *H. indica* SL0708 (n = 20) was evaluated under a completely randomized design. The experiment was repeated three times on different dates. Significant differences were observed (F = 11.127, df = 7. 24, p = 0.0054), registering mortality between 3 and 14 days. *Steinernema* sp3 JCL027 was the strain producing the highest mortality rate (19.3 ± 8 %), followed by *H. bacteriophora* HNI0100 (5.2 ± 9 %). Thus, we evaluated *Steinernema* sp3 JCL0270 using a randomized design at 0, 160, 290, 420, 550, 680, 810 IJs/cm^2^ (n = 12). The experiment was repeated three times on different dates. Significant differences were found among treatments (44 ± 5 %, F = 14.676; df = 6. 21, p = 0.001), with 680 IJs/cm^2^ producing the highest mortality followed by 810 IJs/cm^2^ (22 ± 5 %). In conclusion, this alternative must be further explored in search of pesticide use and cost reduction, in addition to young palm loss in a plantation.

## Background

*Strategus aloeus* L (Coleoptera: Melolonthidae), known as “Little bull” or “chiza” is an oil palm insect pest in Colombia, Venezuela, Guyana, Suriname, Northern Brazil, Ecuador, and Peru (Aldana et al. 2005). In Colombia, Meta and Cesar are the two departments with greatest national palm industry participation (Torres-Carrasco et al. 2013). Oil palm damage is caused generally by the adult male drilling a hole in the ground, with variable length up to 1.5 m, around the young palm’s bulb (younger than 2 years). Later, the male opens a lateral perforation, where after 10 days the female arrives to copulate. In this process, the young oil palm bulb is damaged, including the meristematic tissue causing the palm’s death (Ahumada et al. 1995). Later on, the saprophylagous larvae feed on the rotting wood, especially in infested renewal palm lots. The life cycle has an average duration of 307.8 days (egg: 14.5 days; instar larvae: First instar larva: 24.4 days, Second instar larva: 41.6 days, Third instar larvae: 200.5 days; pupae: 26.8 days). Furthermore, 90 % of this organism’s life cycle is within the larval stages encompassing 267 days (Ahumada et al. 1995; Coto and Saunders 2004).

*Strategus aloeus* is a pest with economic impact; however its control has been limited to insecticide use and residue handling in renewal palm lots (Cipriano et al. 2010; Aldana et al. 2011). Therefore, entomopathogenic nematodes (EPN) have been considered as a potential control agent. *Heterorhabditis* and *Steinernema* EPN offer advantages of mutualistic association with bacteria of the genera *Xenorhabdus* and *Photorhabdus,* respectively (Kaya and Gaugler 1993), high virulence, and rapid action to kill their host. The infective juvenile stage (IJ) does not feed, since it is morphologically and physiologically adapted to survive for long periods within the soil in the absence of its host. In addition, it is capable of parasitizing its host by entering through the mouth, anus, spiracles, cuticle and first generation heterorhabditid hermaphrodite (Shapiro Ilan et al. 2014). Palmas del Cesar is searching to establish *S. aloeus* biological control to reduce environmental pollution due to continued insecticide use, reduce costs, and loss of young palm plants. Therefore, we evaluated EPN isolated from the Colombian Andean region on *S. aloeus* third instar larvae under laboratory conditions.

## Methods

*Strategus aloeus* larvae were collected in oil palm renewal lots in the department of Cesar in “Palmas del Cesar S.A Group” in Corregimiento de Minas, San Martín-Cesar Km. 113. Third instar larvae were used due to their availability in renewal lots in this plantation.

EPN species/strains were obtained from the Colombian Andean region from Centro Nacional de Investigaciones de Café (CENICAFE Agreement 182-2009, Table 1) and Pontificia Universidad Javeriana. All assays were performed under controlled conditions at 15 °C and 75 % relative humidity in the Laboratory for Biological Control.

**Table 1:** Evaluated entomopathogenic nematode (EPNs) species/strains for the control of *Strategus aloeus*

Species	Strain	Source
*Steinernema websteri*	JCL006	Chinchiná—CaldasCenicafe
*Steinernema feltiae*	SCT125	Scientia
*Steinernema* sp3	JCL027	Sasaima—Cundinamarca Cenicafe
*Steinernema colombiense*	SNI0198	Quimbaya—Quindio Cenicafe
*Heterorhabditis bacteriophora*	HASA702	Riofrio—Valle del Cauca
*Heterorhabditis bacteriophora*	HNI0100	Fresno—Tolima Cenicafe
*Heterorhabditis indica*	SL0708	Alcaliá—Valle del Cauca

### Susceptibility evaluation

Oil palm plantation soil (89.5 g) was placed into a plastic vial (10.3 cm × 8.5 cm × 5.0 cm) with one *S. aloeus* larvae and 160 IJs/cm^2^ resuspended in 3 ml dH_2_O (Table 1). Untreated controls were identical to the treatments except that no IJs were added. Larvae mortality was registered every 24 h during 2 weeks, based on symptoms and corroborated by dissections, to verify nematode presence. Based on these results (highest mortality Table 1) different IJs doses were assayed at 0, 160, 290, 420, 550, 680, 810 IJs/cm^2^ in 3 ml dH_2_O suspension to select the best dose response (Grewal et al. 2005).

### Statistical analysis

Susceptibility evaluation was performed with a completely randomized design (7 EPNs isolations *vs.* larval stage of the insect pest). Each treatment consisted of 20 experimental units and the experiment was repeated three times on different dates. For dose evaluation a completely randomized design was used (one EPN species/strain vs. pest larval stage). Each treatment had 12 experimental units and the experiment was repeated three times on different dates.

All data were analyzed for normality (Shapiro–Wilk and Kolmogorov–Smirnov test) and homogeneity of variance (Levene test) with comparisons established by ANOVA. For significant differences between treatments an HSD Tukey test was performed. All analyses were carried out in SPSS-PC v.21 and Statistix 9.0, with a significance level of *p* < 0.05.

## Results

**Fig. 1:**
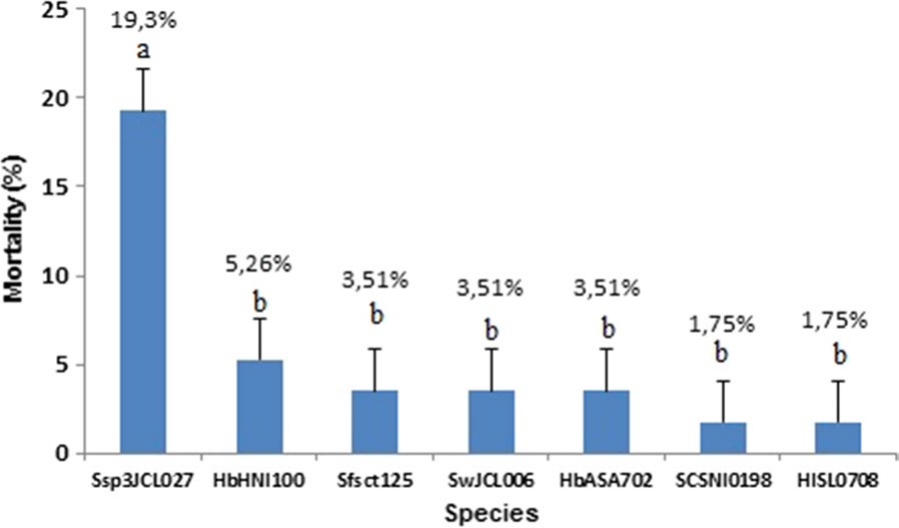
Third instar larvae mortality percentage (Mean ± SEM). *Strategus aloeus* third instar larvae mortality percentage treated with Ssp3JCL027: *Steinernema* sp3 JCL027; HbHNI0100: *H. bacteriophora* HNI0100; Sfsct125: *S. feltiae* SCT125; Sw JCL006: *S. websteri* JCL006; HbASA702: *H. bacteriophora* ASA702; SC SNI0198: *S. colombiense* SNI0198; HI SL0708: *Heterorhabditis indica* SL0708. *Bars* indicate standard deviation. *Letters above bar* indicate significant differences (ANOVA with Tukey post hoc test *p* < 0.05)

*Strategus aloeus* third instar larvae were susceptible to all EPNs species/strains evaluated (F = 11.127, df = 7, 24, p = 0.0054). Mortality was achieved between 3 and 14 days of treatment, with the latter hours presenting the greatest mortality rates. There was no mortality for controls. *Steinernema* sp3 JCL027 was the strain with highest mortality rate (19.3 ± 8 %), followed by *H. bacteriophora* HNI0100 (5.2 ± 9 %). The remaining treatments were between 1.75 and 5.26 % (Fig. 1).

**Fig. 2:**
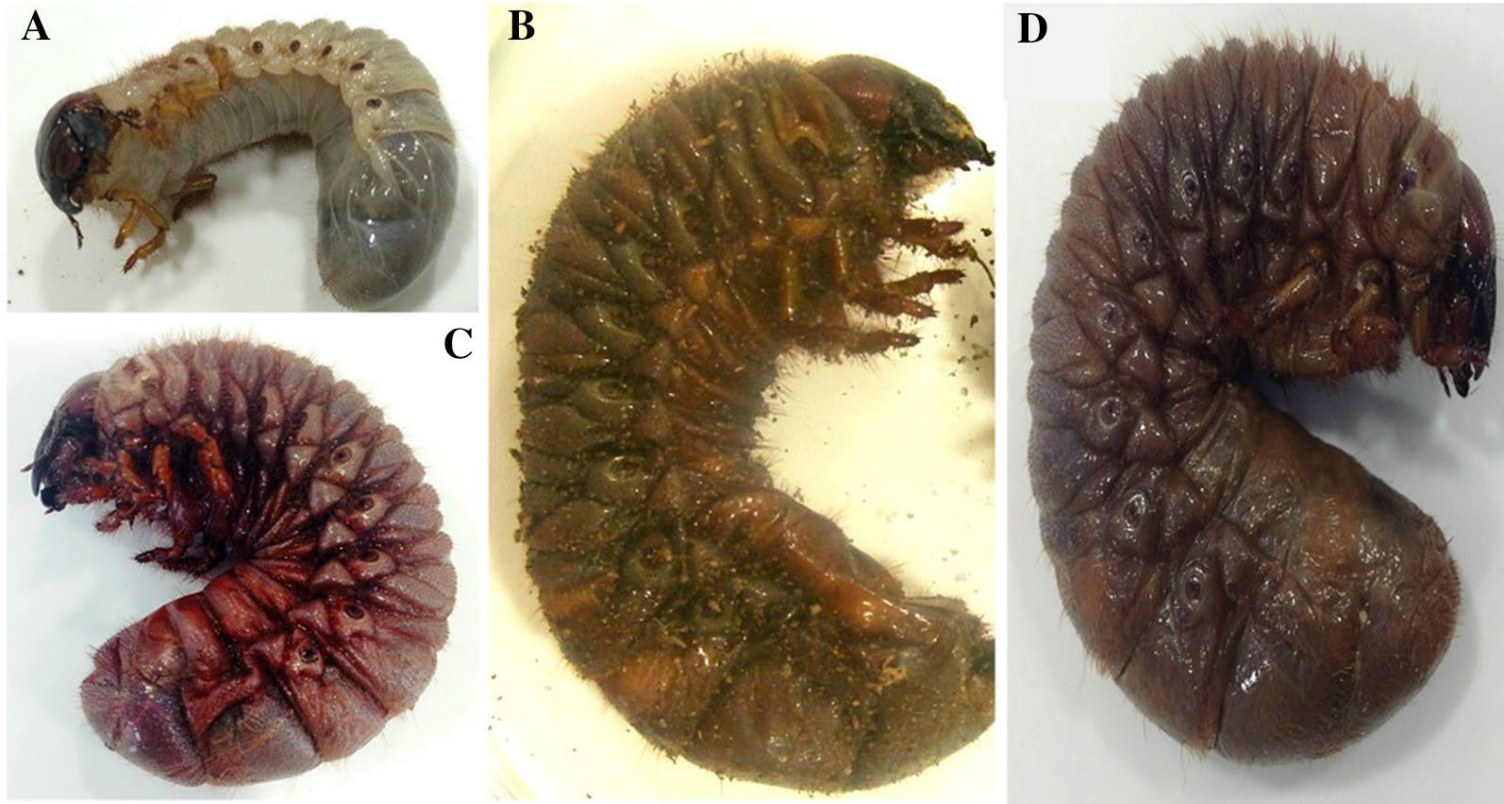
*Strategus aloeus* third instar larvae coloration in control and entomopathogenic nematode treatments: **a** Healthy larvae. **b** Infected larvae with *Heterorhabditis indica* SL0708. **c** Infected larvae with *H. bacteriophora* ASA702. **d** Infected larvae with *Steinernema* sp3 JCL027

Symptoms of susceptibility in *S. aloeus* included red coloration patterns for individuals affected by Heterorhabditidae (Fig. 2a–c) and different browns for Steinernematidae (Fig. 2a, d). With the exception of the posterior segment a sagging body was observed, due to substrate accumulation, with lack of putrefying smell. In addition, total liquefaction of internal tissues was evidenced, as well as presence of nematode developing stages: females, males, hermaphrodites, and juveniles. Furthermore, we observed intact fat bodies in some dead larvae.

**Fig. 3:**
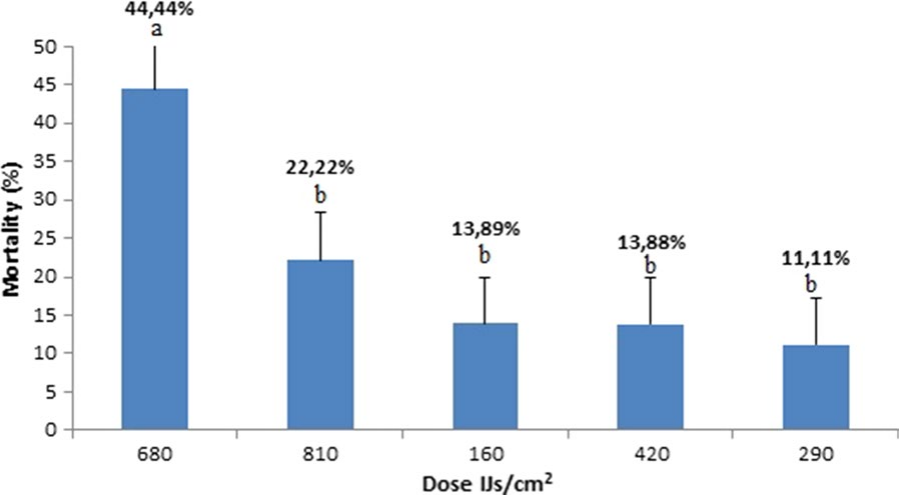
Mortality percentage. *Strategus aloeus* third instar larvae mortality percentage (Mean ± SEM) treated with different doses of *Steinernema* sp3 JCL027. *Letters above bar* indicate significant differences (ANOVA with Tukey post hoc test *p* < 0.05)

*Steinernema* sp3 JCL027 was selected for dose evaluation, since it presented the highest mortality rate for *S. aloeus* larvae in their third instar (Fig. 1). Significant differences were observed for 680 IJs/cm^2^ (44 ± 5 %, p = 0.001), followed by 810 IJs/cm^2^ (22 ± 5 %) (Fig. 3).

## Discussion

Species and their different instar larvae within the Scarabaeidae and Melolonthidae families frequently present highly variable susceptibilities to EPNs action (Grewal et al. 2005; Koppenhöfer et al. 2006; Khatri-Chhetri et al. 2011). Therefore, it is critical to evaluate species/strain EPNs dose to determine which generates the greatest pathogenicity. All evaluated *S. aloeus* isolations demonstrated important variabilities, with mortality rates inferior to those reported for other instar larvae species affected by Steinernematidae and Heterorhabditidae.

In a report presented by Sánchez-Saavedra et al. (2012), employing a 2500 IJs/ml (19.8 IJs/larvae) *H. indica* dose on different *Phyllophaga* spp. (Coleoptera: Melolonthidae), third instar larvae mortality was 46 %. Likewise, Khatri-Chhetri et al. (2011), assessed the pathogenic capability of seven EPN on *Holotrichia longipennis* (Coleoptera: Scarabaeidae) third instar larvae using a concentration of 1000 IJs/larva (50 IJs/cm^2^) with mortalities ranging between 35.6 and 72.8 %. Furthermore, Quintero-Marin et al. (2006) evaluated *H. bacteriophora* HNI0100, *Steinernema* sp. SNI0198, and *Heterorhabditis* spp. pathogenicity and infectivity.

Centro International de Agricultura Tropical (CIAT) conducted a study where doses of 7000 and 13,000 IJ/larvae (7000 and 13,000 IJs/ml) with both *Steinernema* and *Heterorhabditis* as a biological insecticide in *Phyllophaga menetriesi* (Coleoptera: Melolonthidae) third instar pest management was tested. Their results evidenced a greater infectivity for *Steinernema* genus (>80 %) in comparison with *Heterorhabditis* (52.9 %). Melo et al. (2007) evaluated *S. feltiae* and *H. bacteriophora* (HNI) (10,000 IJs/ml–10,000 IJs/larvae) pathogenicity on *P. menetriesi* and *Anomala inconstans* (Coleoptera: Melolonthidae) third instar larvae. Their results demonstrated greater mortalities for *H. bacteriophora* HNI.

Contrary to that reported in the literature, S*teinernema* sp3 JCL027 had a greater susceptibility compared with *Heterorhabditis* efficacy against *S. aloeus* third instar larvae. We suggest S*teinernema* sp3 JCL027 IJs were able to overcome the host’s barriers and physiological mechanisms to evade an immune response or digestive tract acidity, permitting the subsequent nematode-bacteria infective cycle development (An et al. 2012; Demir et al. 2014). Taking into account Scarabaeidae and Melolonthidae immune response defense, in particular during their third instar (Griffin et al. 2005), we expected *S. aloeus* would be more susceptible to *Heterorhabditis* than Steinernematids based on certain advantages (Khatri-Chhetri et al. 2011; Vashisth et al. 2013). As a case in point, *Heterorhabditis* IJs can enter its host through intersegmental membranes of the cuticle. Moreover, it exhibits a foraging behavior depending on the species to be controlled. However, Scarabaeidae and Melolonthidae third instar larvae present a thicker cuticle (Rodríguez et al. 2009), possibly impeding intersegmental membrane access. Therefore, this event may account for lower *S. aloeus* mortality rate when using *Heterorhabditis* as a pest control.

Under controlled laboratory conditions, during our nematode species/strain screening, *S. aloeus* mortality rate did not exceed 20 % compared with mortality rates of 46 to 85 % for other reports in the literature (Grewal et al. 2005; Quintero-Marin et al. 2006; Melo et al. 2007). We suggest our result might be due *S. aloeus* constant grooming behavior by scrubbing its raster with the aid of its legs and mouth parts. In addition, it is capable of rapid mandible movement, evasive behavior, and constant defecation. Probably, *S. aloeus* significantly reduced its susceptibility to each of the EPNs isolates it was exposed to. Another factor affecting susceptibility variation could be EPNs-host specificity. Larvae could have been partially resistant to EPNs’ infection, due to *S. aloeus* morphology, physiological defense mechanisms or IJs symbiotic bacteria. This latter one could have possibly not been virulent for this insect pest, as it has been reported for other Lepidoptera (Bisch et al. 2015).

It was not unexpected that an increased *Steinernema* sp3. JCL027 concentration did not always present a concomitant increase in *S. aloeus* death, as was the case for the 680 IJs/cm^2^ and 810 IJs/cm^2^ doses. Quintero-Marin et al. (2006) evaluated the pathogenicity and infectivity of three EPNs, finding similar results. In their study *Steinernema* spp. SNI 0198 and *H. bacteriophora* HNI 0100 presented a greater infectivity (% penetration) at a lower dose (7000 IJs), while the 13,000 dose was significantly lower. Likewise they found in their first pathogenicity reading *Steinernema* sp. SNI 0198 and *Heterorhabditis* spp. CIAT also presented greater mortalities at the lower does (7000 IJs/ml compared with the 13,000 dose). According to Kaya and Koppenhöfer (1996), this can be attributed to IJs intraspecific competition to enter the host at a given concentration. It is important to keep in mind that exposing a host to specific numbers of nematodes does not imply that all of them can enter the host. Even if they are capable of doing so, they still have to encounter a number of barriers until reaching the hemocoel. Furthermore, penetration does not necessarily imply a subsequent mortality (Demir et al. 2014; Bisch et al. 2015).

Observed symptoms in *S. aloeus* third instar larvae after performed infections agree with what has been described for other larvae of the Scarabaeidae and Melolonthidae families: larvae stop eating; they decrease their motility, and change in color (Rodríguez et al. 2009; Sánchez-Saavedra et al. 2012). However, when we performed dissections to validate mortality not all were found to contain liquefied tissues. The life cycle of the infective juvenile requires the nematode to penetrate the host, and then enter into the hemocoel. The juvenile can then release their symbiotic bacteria from their intestines into the host’s hemocoel. The bacteria can subsequently proliferate in the host causing septicemia, permitting the nematode to develop its life cycle. (An et al. 2012; Bisch et al. 2015). Collectively, reports describe some bacteria are capable of eliciting a pathogenic reaction, therefore the host’s immune response acts by encapsulation, phagocytosis, among others. In this manner the nematode life cycle does not develop. Even though these events have been characterized we did not evaluate them, therefore we cannot assume they are associated.

## Conclusion

To achieve even higher *S. aloeus* larvae mortality percentages than was obtained in the “Palmas del Cesar” management program using the tested EPNs as biological control agents, it may be pertinent to evaluate other entomopathogenic fungi including *Beauveria bassiana* and *Metarhizium anisopliae* naturally affecting the third instar larvae, as well as *Serratia* spp. an entomopathogenic bacteria. In addition, synergistic associations may occur among combinations of different entomopathogens. Also, it would be worthwhile to evaluate virulent capability of other EPNs (individual or combined applications) adapted for these larvae, possibly present in oil palm lots and their symbiotic bacteria for all instar larvae and adult *S. aloeus.*
